# Optimizing Cost-Effective Larval Diets for Mass Rearing of *Aedes* Mosquitoes in Vector Control Programs

**DOI:** 10.3390/insects16050483

**Published:** 2025-05-01

**Authors:** Qianqian Li, Tongxin Wei, Yan Sun, Jehangir Khan, Dongjing Zhang

**Affiliations:** 1 Clinical Medicine, Zhongshan School of Medicine, Sun Yat-sen University, Guangzhou 510080, China; 2 Chinese Atomic Energy Agency Center of Excellence on Nuclear Technology Applications for Insect Control, Key Laboratory of Tropical Disease Control of the Ministry of Education, Sun Yat-sen University, Guangzhou 510080, China; 3 International Atomic Energy Agency Collaborating Centre, Sun Yat-sen University, Guangzhou 510080, China; 4 Guangdong Provincial Engineering Technology Research Center for Diseases-Vectors Control, Sun Yat-sen University, Guangzhou 510080, China; 5 Department of Zoology, Abdul Wali Khan University, Mardan 23200, Pakistan; 6 Hainan General Hospital, Hainan Medical University, Haikou 570100, China

**Keywords:** larval diets, *Ae. aegypti*, *Ae. albopictus*, life history traits, mass rearing

## Abstract

(1) Background: Larval diet composition significantly influences the developmental, physiological, and reproductive traits of *Ae. albopictus* and *Ae. aegypti*, major arbovirus vectors. Optimizing larval nutrition is essential for mass-rearing programs supporting the sterile insect technique and incompatible insect technique. This study evaluated the effects of three larval diets on key fitness traits, including pupation rate, male flight ability, adult longevity, female fecundity, pupal size, and wing length, which are critical for the success of SIT and IIT programs. (2) Methods: *Ae. albopictus* (GT strain) and *Ae. aegypti* (AEG strain) were reared on three diets with varying protein sources: diet 1 (≈1.23 dollars/kg; porcine liver/shrimp/yeast = 6:3:1), the IAEA-recommended diet; diet 2 (≈1.78 dollars/kg; bovine liver/shrimp/yeast = 6:3:1), a modified IAEA diet; and diet 3 (≈0.55 dollars/kg; tortoise food), a low-cost laboratory formulation. Life history traits were assessed using standardized protocols, and data were analyzed with ANOVA and Tukey’s post hoc test. (3) Results: Diet 3 consistently improved pupation rates, adult longevity, and male flight ability compared with diet 2. Mosquitoes reared on diets 1 and 3 exhibited significantly larger pupae and longer wings, while diet 2 performed sub-optimally. Adult eclosion rates (~100%) remained high across all diets. Male flight ability varied by species, with *Ae. albopictus* performing best on diet 1 and *Ae. aegypti* on diet 3. Female fecundity was diet-dependent, with diet 1 favoring *Ae. albopictus* and diet 3 benefitting *Ae. aegypti*. Longevity was highest in mosquitoes reared on diet 3, with a median survival of 19.5 days for GT males and 37.5 days for GT females. (4) Conclusions: Diet 3 emerged as the most cost-effective option, enhancing key fitness traits essential for SIT and IIT. Future studies should refine nutrient formulations and validate findings under field conditions to optimize mass-rearing efficiency in vector control.

## Simple Summary

Mosquitoes such as *Aedes albopictus* and *Ae. aegypti* are major vectors of diseases like dengue and Zika, posing significant threats to global public health. To combat these mosquitoes, methods like the sterile insect technique (SIT) and incompatible insect technique (IIT) are used, which rely on releasing large numbers of sterilized or modified males to suppress wild populations. However, the success of these programs depends on producing high-quality mosquitoes in laboratory settings, which requires optimized nutrition during the larval stage. This study investigated the role of larval diets in optimizing mosquito fitness for mass-rearing programs. Three diets with varying costs and protein sources were tested to identify the most effective option for large-scale rearing. The results indicated that a low-cost diet based on tortoise food improved mosquito survival, flight ability, and longevity, making it a promising alternative for cost-effective production. By identifying effective larval diets, this research contributes to improving mosquito mass-rearing practices, which are critical components of SIT and IIT operations. Future studies will focus on refining these diets and validating their effectiveness in the real world, supporting global efforts to combat mosquito-borne diseases.

## 1. Introduction

Mosquitoes (Diptera: Culicidae) serve as vectors for numerous human and zoonotic diseases, including malaria, dengue fever, Zika virus, filariasis, and viral encephalitis, posing a significant threat to global public health [1]. With over 3600 mosquito species classified into 39 genera and 135 subgenera, certain mosquitoes play a critical role in transmitting life-threatening pathogens [1,2]. Among these, *Aedes* mosquitoes are particularly important as they are the primary vectors of the dengue virus (DENV), which has experienced a 30-fold increase in incidence over the past 50 years. Today, dengue affects over 400 million people annually, highlighting the urgent need for effective control measures [3].

Traditional mosquito control strategies, such as insecticide use, have faced significant challenges, including the development of resistance, adverse effects on non-target species, and environmental harm [4]. These limitations have driven the development of alternative, more sustainable approaches. Among these, the sterile insect technique (SIT) and incompatible insect technique (IIT) have emerged as promising, species-specific solutions. Both techniques involve the release of mass-reared males that are either sterilized or infected with Wolbachia bacteria, thereby reducing their reproductive potential and progressively suppressing wild populations [5].

The success of SIT and IIT hinges on the production of high-quality, competitive males that exhibit traits comparable to their wild counterparts, including physiological fitness, flight ability, mating success, and sufficient longevity for multiple mating opportunities [5,6]. Additionally, the productivity of rearing systems depends on female fecundity, which ensures a consistent supply of males. Among the factors influencing mass-rearing success, larval diet plays a pivotal role in shaping adult phenotypic traits such as survival, fitness, and reproductive potential [1]. Optimal larval nutrition, incorporating balanced macronutrients (carbohydrates, proteins, and lipids), is essential for producing robust adults capable of surviving and reproducing in the wild [7,8].

Current commercial diets, often formulated with high-cost ingredients such as brewer’s yeast or liver formulations, are impractical for large-scale operations, particularly in resource-limited settings [9,10]. For instance, diets recommended by the International Atomic Energy Agency (IAEA) [11] and Guangzhou Wolbaki Biotech Co., Ltd. (Guangzhou, China), can cost up to USD 18–33 per kilogram, posing significant challenges for widespread applications [6]. Thus, identifying cost-effective, locally sourced, and nutritionally balanced alternatives is critical for supporting large-scale mosquito rearing efforts.

This study addressed this gap by evaluating three alternative larval diets and their effects on the developmental and physiological traits of *Ae. albopictus* and *Ae. aegypti*. The tested diets included (1) the IAEA diet (bovine liver, shrimp, and yeast powder), (2) an IAEA-modified pork liver-based diet, and (3) a tortoise food-based diet. Key life history traits, such as development time, survival, wing size, fecundity, and longevity, were assessed. By optimizing larval nutrition, this research aimed to enhance the competitiveness and quality of male mosquitoes, ultimately supporting the development of more sustainable and scalable vector control strategies for combating dengue and other mosquito-borne diseases.

## 2. Materials and Methods

### 2.1. Mosquito Strains

Two *Aedes* mosquito species were used in this study. The *Ae. albopictus* GT strain, an aposymbiotic line developed in our lab [12], was selected for its demonstrated comparability to wild-type strains in fitness traits and its successful use in field suppression trials [5,13]. Its *Wolbachia*-free status also serves as a reliable genetic marker for distinguishing released males from wild populations, facilitating accurate monitoring of the release ratio in SIT programs [5,13]. The *Ae. aegypti* (recorded as AEG) was provided by the Guangdong Provincial Center for Disease Control and Prevention (Guangzhou, China). This choice aligned with the naturally *Wolbachia*-free state of *Ae. aegypti* in field populations, enabling us to isolate dietary effects on life history traits without confounding influences from *Wolbachia*. These two mosquito strains have been maintained for over 10 years in a climate-controlled insectary room at 27 ± 1 °C, 80 ± 10% RH, and a photoperiod of 12:12 (L:D) h in the laboratory of the Zhongshan School of Medicine, Sun Yat-sen University (Guangzhou, China).

### 2.2. Study Design

**Figure 1:**
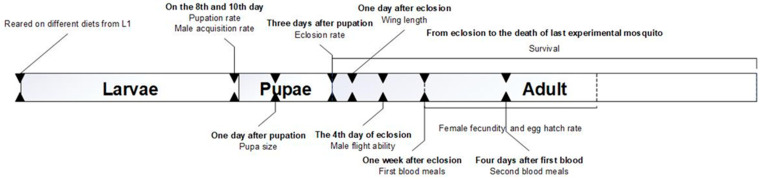
A conceptual framework of the experiment.

Three larval diets were assessed in a randomized experimental design with 3–4 replicates per diet, each containing 100 newly emerged L1 larvae of *Ae. albopictus* (GT strain) or *Ae. aegypti* (AEG strain). Each replicate was reared independently in a separate tray under controlled conditions. Diet 1, the IAEA-recommended food, was chosen as the reference (control) diet due to its proven efficacy in the mass rearing of mosquito species, as demonstrated previously [14]. Diet 2 was a modified version of diet 1 [12], while diet 3, commercially available tortoise food, was adapted for mosquito larval rearing in this study. Figure 1 illustrates the experimental framework, with detailed procedures in subsequent sections.

### 2.3. Formulation and Characteristics of Specialized Diets

Table 1 provides an overview of the ingredients and nutritional composition of the three larval diets assessed in this study. Diet 1 and diet 2 were formulated using pork liver, bovine liver, shrimp, and yeast powders in specific proportions, while diet 3 comprised commercially available tortoise food. These diets differed in price, color, texture, and key nutritional properties. Feeding was initiated at the L1 stage. Nutritional analyses were conducted to evaluate the essential components for mosquito development, aiming to identify cost-effective and nutritionally balanced diets suitable for mass-rearing programs.

### 2.4. Rearing Conditions

**Figure 2:**
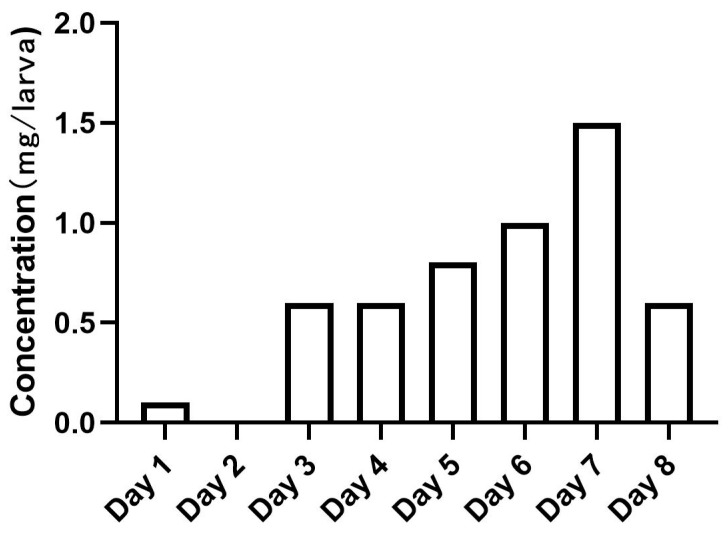
Daily diet supply for *Ae. albopictus* and *Ae. aegypti* larval rearing.

Eggs from both GT and AEG strains were hatched in plastic trays (dimensions: 17 cm × 11 cm × 7 cm) containing approximately 0.7 L of deionized water. Two to four hours post-hatching, 100 newly emerged larvae (L1) were pipetted into circular plastic trays (top diameter, 11 cm; bottom diameter, 7.5 cm; height, 6 cm) containing 150 mL of deionized water with added larval diet. Daily diet quantities for diets 1, 2, and 3, all dry materials, were fixed at 0.1–1.5 mg per larva, following a predetermined schedule (Figure 2) informed by Zhang et al. [15] and laboratory optimization, to ensure consistent, repeatable delivery across treatments and minimize bias from differential pupation rates. The larvae were maintained in an artificial climate chamber (BPH-9082, Shanghai Yiheng Technology Co., Ltd., Shanghai, China) at 27 °C and 80% relative humidity. To minimize evaporation, the trays were covered with lids, and the water levels were replenished as needed. Adults were kept in standard 30 × 30 × 30 cm plastic cages in the climate-controlled room at 27 ± 1 °C, 80 ± 10% RH, and a photoperiod of 12:12 (L:D) h and were continuously supplied with a 10% sugar solution.

### 2.5. Pupation Rate and Male Acquisition Rate

The appearance of the first pupa was recorded approximately on day 6 post-L1. Pupae were collected and counted daily until day 10 post-L1, by which time nearly all larvae had pupated. Pupal sex was determined under a stereomicroscope (SZ650, Chongqing Optec Instrument Co., Ltd., Chongqing, China). For each diet, four replicates were used, with 100 L1 larvae per replicate for each strain of mosquito. The pupation rate was calculated as the number of pupae per replicate on day 8 divided by the initial number of larvae (*n* = 100), reported separately for males and females. Day 8 was selected as the primary reference point as most larvae had pupated by then, although rates were also calculated on day 10 for completeness. The pupal sex ratio (% male) was calculated as the mean proportion of male pupae among total pupae per replicate on days 8 and 10, reflecting observed ratios rather than assuming a 1:1 larval sex ratio, which required batch-specific validation [12]. This approach avoided theoretical assumptions about male yield, aligning with prior work [8], and provided an empirical measure of diet effects on pupal sex distribution. As SIT and IIT programs rely on sex separation at the pupal stage, these data indicated which diets may support higher male pupal production, a key factor in rearing optimization.

### 2.6. Pupal Size

From three replicates, 10–20 pupae per sex per diet (total 20–40 per diet) were randomly selected. Pupae were treated at −20 °C for 5 min to restrict their mobility. The samples were placed on a 96-well plate with icy water. Then a digital image of the pupa cephalothorax in tergal view was made using a camera mounted on a stereomicroscope, and the actual measurement was performed using the analysis Mshot software version 1.1.6 A (Guangzhou TWS Electronics Co., Ltd., Guangzhou, China), whose precision was ±0.0001 mm. The pupal size was determined by measuring the width of the cephalothorax at its widest point in tergal view [16], with measurements reported separately for males and females.

### 2.7. Eclosion Rate

After segregating male and female pupae during the pupal stage, a total of 30 male and 30 female pupae were randomly selected from each of three replicates and transferred into circular plastic cups (top diameter, 6 cm; bottom diameter, 5.5 cm; height, 4.5 cm). Each cup contained approximately 80 mL of deionized water. The cups were then placed within stainless steel cages (30 × 30 × 30 cm). Adult eclosion was monitored daily for three consecutive days post-pupation. The eclosion rate was calculated as the number of successfully emerged adults divided by the total number of pupae (eclosion rate = emerged adults/total pupae), as described previously [14].

### 2.8. Wing Length

Three days post-eclosion, 10 adults per sex per diet (total 20 per diet) were randomly selected from three replicates. They were immobilized at −20 °C for 5 min. Wing length was measured (±0.001 mm) from the proximal base to the radial vein’s distal end using Mshot software [12]. Mean values per replicate were used for analysis.

### 2.9. Male Flight Ability

To assess male flight ability, around 1000 eggs from both the GT and AEG strains were hatched in plastic trays (dimensions, 50 cm × 40 cm × 4 cm) containing approximately 7 L of deionized water. The daily diet quantities were dispensed according to the concentration shown in Figure 2. After the larvae pupated, male and female pupae were separated during the pupal stage, and male pupae were transferred into stainless steel cages (30 × 30 × 30 cm). Five to six days post-eclosion, 50–150 male adults (per replicate) were introduced into the flight test device (FTD), a cylindrical apparatus (height, 30 cm; diameter, 10 cm), using an aspirator, following the described method [16]. The introduced mosquitoes must fly upward through the 40 flight tubes (each 25 cm high with an 8 mm inner diameter) to escape from the device. The males were given a two-hour period to escape through the flight tubes. After two hours, the entire device was placed at −20 °C for approximately 30 min to immobilize the adults. The number of males remaining at the base of the flight tubes and those that successfully escaped were counted. Male flight ability was assessed by calculating the escape rate, defined as the ratio of the number of adults that had successfully escaped to the total number of adults introduced into the flight tube. Each treatment was replicated three times.

### 2.10. Female Fecundity and Egg Hatch Rate

About 50 male and 150 female pupae from the three replicates were grouped and caged in a 30 × 30 × 30 cm cage [12]. Seven days post-eclosion, females were provided with blood meals via mouse feeding. Ten to twenty engorged females fed on different diets were individually placed in plastic cups (110 mL) lined with moist filter paper for oviposition [4]. Cups were maintained under standard laboratory conditions (27 ± 1 °C, 80 ± 10% RH, 12:12 L:D photoperiod). A second blood meal was provided four days after the first to assess fecundity. Eggs were incubated for seven days to ensure maturation, after which fecundity (total eggs laid per female) and egg hatch rate (hatched eggs/total eggs per female, expressed as a percentage) were determined.

### 2.11. Survival

To evaluate adult longevity, 30 males and 30 females, newly emerged, were housed together in 15 × 15 × 15 cm stainless steel cages at 27 ± 1 °C, 80 ± 10% RH, and a photoperiod of 12:12 (L:D) h [12]. Both sexes were provided constant access to a 10% sucrose solution, which was replenished daily to ensure a consistent energy source. Mortality was recorded daily by inspecting the cages and removing dead individuals. Observations continued until all individuals in the cohort had died, with the survival time for each mosquito noted. The survival data were analyzed to compare male and female longevity as previously described [12].

### 2.12. Statistical Analysis

The statistical analysis was conducted using the GraphPad Prism 6.0 software. Data normality and variance homogeneity were assessed with D’Agostino–Pearson and Brown–Forsythe tests. One-way ANOVA and Tukey’s post hoc test were used to compare the effects of the 3 larval diets on the pupation rate, male pupal recovery, eclosion rate, pupal size, wing length, fecundity, and egg hatch rate. Survival was analyzed with Kaplan–Meier curves and log-rank tests (*p* < 0.05).

## 3. Results

### 3.1. Pupation Rate

The pupation rates of *Ae. albopictus* (GT) and *Ae. aegypti* (AEG) varied significantly across larval diets on days 8 and 10 post-L1 (Table 2). For GT, diets 1 and 3 consistently yielded higher pupation rates than diet 2 for both sexes (day 8, males, *F =* 15.96, *d.f.* 2, 9, *p* = 0.0011; females, *F =* 53.42, *d.f.* 2, 9, *p* < 0.0001; day 10, males, *F =* 8.295, *d.f.* 2, 9, *p* = 0.0091; females, *F =* 43.38, *d.f.* = 2, 9, *p* < 0.0001), with no significant difference between diets 1 and 3. For AEG, diet 3 produced the highest rates, followed by diet 1, with diet 2 consistently lowest (day 8, males, *F =* 30.44, *d.f.* 2, 9, *p* < 0.0001; females, *F =* 10.91, *d.f.* 2, 9, *p* = 0.0039; day 10, males, *F =* 66.15, *d.f.* 2, 9, *p* < 0.0001; females, *F =* 42.41, *d.f.* 2, 9, *p* < 0.0001); diets 1 and 3 differed significantly only for males on day 8 (*p* < 0.05). These results, consistent across species and sexes, highlight a strong dietary influence on developmental progress (see Table 2 for detailed rates).

### 3.2. Male Sex Ratio

The male sex ratio for *Ae. albopictus* (GT) differed significantly across larval diets on days 8 and 10 post-L1 (day 8, *F* = 10.80, *d.f.* 2, 9, *p* = 0.0041; day 10, *F* = 12.54, *d.f.* 2, 9, *p* = 0.0025) (Table 3), while there was no difference for *Ae. aegypti* (AEG). For GT, diet 2 outperformed diets 1 (day 8, *p* < 0.05; day 10, *p* < 0.05) and 3 (day 8, *p* < 0.05; day 10, *p* < 0.05). Because male larvae generally develop faster than females, the higher male sex ratio observed under diet 2 in GT suggested that larvae under this diet reached pupation more quickly than those under diets 1 and 3, indicating relatively faster growth.

### 3.3. Pupal Size and Wing Length

**Figure 3:**
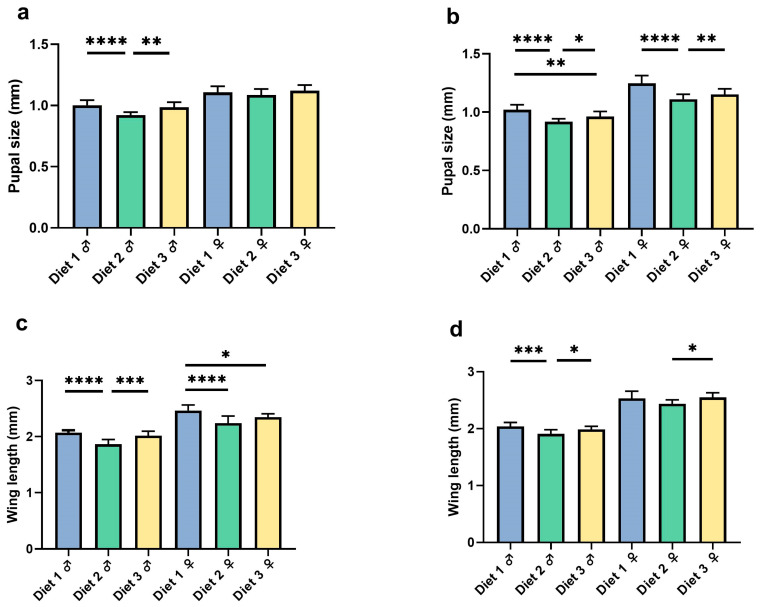
Pupal size (**a**,**b**) and wing length (**c**,**d**) of *Ae. albopictus* (GT) and *Ae. aegypti* (AEG) across diets. Bars represent means ± SD of pupae (**a**,**b**) or adults (**c**,**d**). Asterisks denote a statistically significant difference using ANOVA analysis and Tukey post hoc test. **** indicates *p* < 0.0001; *** indicates *p* < 0.001; ** indicates *p* < 0.01; * indicates *p* < 0.05.

The pupal size and wing length of *Ae. albopictus* (GT) and *Ae. aegypti* (AEG) varied significantly across larval diets, with diets 1 and 3 generally surpassing diet 2 (Figure 3).

#### 3.3.1. Pupal Size

The pupal size varied significantly across diets, except for GT female (GT male, *F =* 15.44; *d.f.* 2, 37; *p* < 0.0001; GT female, *F =* 1.414; *d.f.* 2, 39; *p* < 0.25; AEG male, *F =* 20.50; *d.f.* 2, 30; *p* < 0.0001; AEG female, *F =* 17.30; *d.f.* 2, 27; *p* < 0.0001). The pupal size was significantly larger for males reared on diets 1 and 3 compared with diet 2 in both GT (*p* < 0.0001) and AEG (*p* < 0.0001) and female AEG (*p* < 0.0001) (Figure 2). However, no significant differences were observed between diets 1 and 3 except for male AEG, where the pupal size was significantly larger under diet 1 (1.02 ± 0.04 mm on diet 1 vs. 0.96 ± 0.04 mm on diet 3; *p* = 0.0035). In AEG, females also exhibited significantly (*p* < 0.0001) larger pupae on diets 1 (1.25 ± 0.07 mm) and 3 (1.15 ± 0.05 mm) compared with diet 2. No significant differences in female pupal size were observed between diets 1 and 3.

#### 3.3.2. Wing Length

The wing length differed significantly across diets (GT male, *F =* 23.14; *d.f.* 2, 27; *p* < 0.0001; GT female, *F =* 13.04; *d.f.* 2, 27; *p =* 0.0001; AEG male, *F =* 9.573; *d.f.* 2, 27; *p =* 0.0007; AEG female, *F =* 3.952; *d.f.* 2, 27; *p =* 0.0312). Male wing lengths were significantly (*p* = 0.0007) larger for both GT and AED under diets 1 and 3 compared with diet 2. GT males exhibited mean wing lengths of 2.07 ± 0.04 mm (diet 1), 2.02 ± 0.08 mm (diet 3), and 1.86 ± 0.08 mm (diet 2) (*p* < 0.0001). Likewise, AEG males showed mean wing lengths of 2.04 ± 0.07 mm (diet 1), 1.99 ± 0.06 mm (diet 3), and 1.91 ± 0.07 mm (diet 2) (*p* = 0.0007). No significant differences were found between diets 1 and 3 for either species. Moreover, the wing length in females was larger under diets 1 and 3 compared with diet 2. For GT females, significant differences were observed between diets 1 (2.47 ± 0.10 mm) and 2 (2.24 ± 0.13 mm; *p* < 0.0001) but not between diets 3 (2.35 ± 0.06 mm) and 2 (*p* = 0.0528). In AEG females, wing lengths were significantly larger on diet 3 (2.55 ± 0.08 mm) compared with diet 2 (2.44 ± 0.07 mm; *p* = 0.0369), while diet 1 (2.53 ± 0.13 mm) was not significantly different from diet 2 (*p* = 0.0900). These results indicate that diets 1 and 3 consistently promoted superior growth in terms of pupal size and wing length compared with diet 2. This effect was particularly strong in males, whereas the significance levels for females varied across comparisons.

### 3.4. Eclosion Assessment

**Figure 4:**
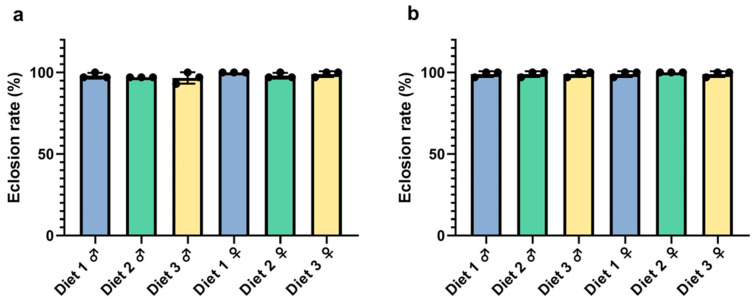
Eclosion rates of GT (**a**) and AEG (**b**) reared on different larval diets. Each bar represents mean ± SD.

The eclosion rates of *Ae. albopictus* and *Ae. aegypti* reared on different diets were consistently high across all treatments, with no significant differences observed among diets 1, 2, and 3 for either species or sex (Figure 4) (GT male, *F =* 0.2826; *d.f.* 2, 6; *p =* 0.7633; GT female, *F =* 1.500; *d.f.* 2, 6; *p =* 0.2963; AEG male, *F =* 1.000; *d.f.* 2, 6; *p =* 0.4219; AEG female, *F =* 0.5000; *d.f.* 2, 6; *p =* 0.6297). For both GT (Figure 4a) and AEG (Figure 4b), the eclosion rates remained near 100% across experimental groups (*p* > 0.05). These results indicated that larval diet composition did not significantly affect pupal eclosion under the tested conditions. Overall, all three diets were equally effective in supporting adult emergence, demonstrating robustness in eclosion success across dietary variations.

### 3.5. Male Flight Ability

**Figure 5:**
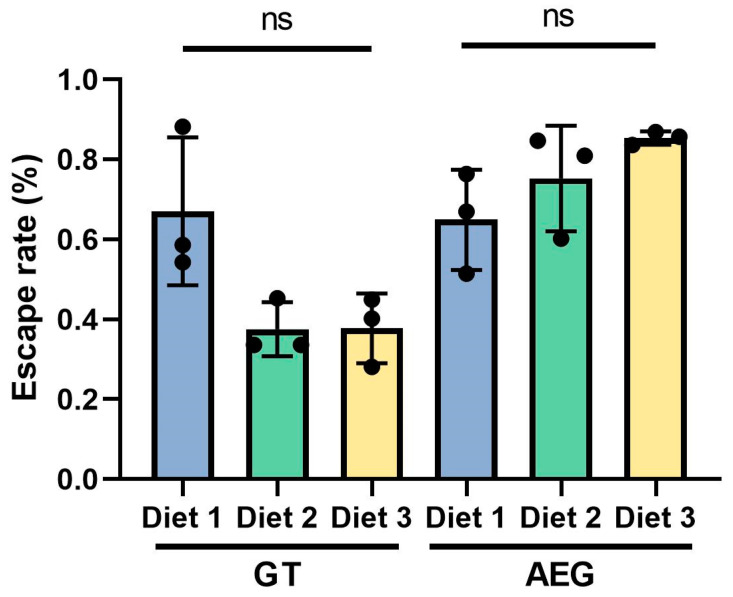
Escape rates of male GT and AEG mosquitoes reared on different diets. Each bar represents mean ± SD. ns denotes a statistically significant difference using ANOVA analysis and Tukey post hoc test. ns indicates *p* > 0.05.

Escape rates of male GT and AEG varied across the different larval diets (Figure 5). In GT males, the escape rates significantly varied among diets (*F =* 5.613; *d.f.* 2, 6; *p* = 0.0423). Males reared on diet 1 exhibited the highest escape rate (0.67 ± 0.18), while diets 2 (0.38 ± 0.07) and 3 (0.38 ± 0.07) showed comparatively lower rates. Pairwise comparisons revealed no significant differences between either of them. In AEG, escape rates were highest in males reared on diet 3 (0.85 ± 0.02), followed by diet 2 (0.75 ± 0.13) and diet 1 (0.65 ± 0.13). However, these differences were not statistically significant (*F =* 2.813, *d.f.* 2, 6, *p* = 0.1374). Overall, diet composition had no effect on male flight ability in *Ae. aegypti*, although an effect was observed in *Ae. albopictus.* The findings indicated that diet composition influenced the flight ability of male mosquitoes, with diet 1 being most favorable for GT and diet 3 showing the greatest benefit for AEG.

### 3.6. Female Fecundity and Egg Hatch Rate

**Figure 6:**
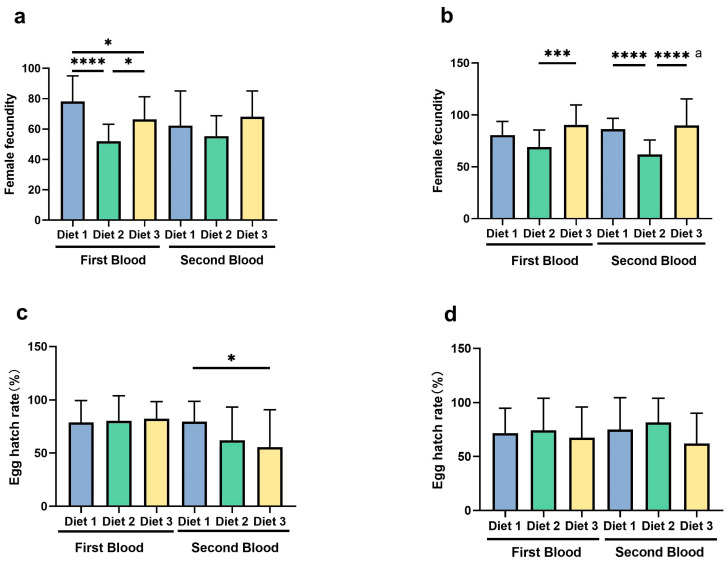
Female fecundity (**a**,**b**) and egg hatch rates (**c**,**d**) of *Ae. albopictus* and *Ae. aegypti* across diets. Bars show means ± SD of 10–30 females after two blood meals (BMs). Asterisks denote a statistically significant difference using ANOVA analysis and Tukey post hoc test, except in a (second blood meal, *Ae. aegypti*), where Brown–Forsythe ANOVA and Games–Howell’s multiple comparisons test were used due to variance heterogeneity. **** indicates *p* < 0.0001; *** indicates *p* < 0.001; * *p* < 0.05.

The analysis revealed significant variations in female fecundity and egg hatch rates of GT and AEG mosquitoes reared on different larval diets (Figure 6). Female fecundity varied significantly following the first blood meal (GT, *F =* 18.57, *d.f.* 2, 63, *p* < 0.0001; AEG, *F =* 9.874; *d.f.* 2, 64; *p* = 0.0002). After the second blood meal, a significant effect was observed in AEG (*F* = 19.19; *d.f.* 2, 61; *p* < 0.0001) but not in GT (*F* = 1.507, *d.f.* 2, 53, *p* = 0.2310). Egg hatching rate showed no significant differences after the first blood meal (GT, *F =* 0.1703, *d.f.* 2, 63, *p* = 0.8438; AEG, *F* = 0.3720, *d.f.* 2, 64, *p* = 0.6908). However, a significant effect was observed after the second blood meal in GT (*F* = 4.028, *d.f.* 2, 53, *p* = 0.0235) but not in AEG (*F* = 2.822, *d.f.* 2, 61, *p* = 0.0673).

#### 3.6.1. Female Fecundity

For GT (Figure 6a), after the first blood meal (BM), fecundity was significantly higher for females reared on diet 1 (78.24 ± 16.80 eggs/female) compared with diets 2 (51.95 ± 11.30 eggs/female) and 3 (66.35 ± 14.96 eggs/female) (*p* < 0.05). However, no significant differences were observed among the diets following the second blood meal (*p* = 0.2310). For AEG (Figure 6b), females reared on diet 3 exhibited the highest fecundity after both the first (90.43 ± 19.17 eggs/female) and second (89.75 ± 25.68 eggs/female) blood meals, significantly outperforming females reared on diet 2 (first BM, 69.12 ± 16.31; second BM, 61.75 ± 14.02) (*p* < 0.05). Females reared on diet 1 displayed intermediate fecundity levels.

#### 3.6.2. Egg Hatch Rates

For GT (Figure 6c), no significant differences in egg hatch rates were observed among the diets following the first blood meal (diet 1, 78.83 ± 20.56%; diet 2, 80.40 ± 23.56%; diet 3, 82.47 ± 15.95%). However, after the second blood meal, diet 1 produced significantly higher hatch rates (79.50 ± 19.20%) compared with diet 3 (55.60 ± 35.16%) (*p* < 0.05). For AEG (Figure 6d), no significant differences in egg hatch rates were observed among the diets after either blood meal (*p* = 0.0673). For the first blood meal, the hatch rates were as follows: diet 1 (71.65 ± 23.10%), diet 2 (74.46 ± 29.62%), and diet 3 (67.52 ± 28.39%). After the second blood meal, hatch rates were highest in diet 2 (81.64 ± 22.42%), followed by diet 1 (75.10 ± 29.52%) and diet 3 (62.13 ± 28.09%).

### 3.7. Longevity Analysis

**Figure 7:**
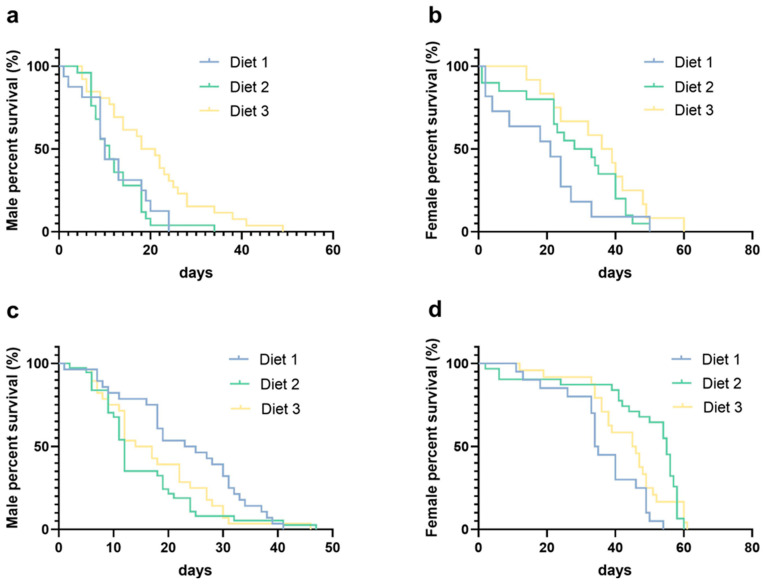
Effects of larval diets on adult survival of *Ae. albopictus* and *Ae. aegypti*. (**a**) GT males, (**b**) GT females, (**c**) AEG males, and (**d**) AEG females. Day number indicates time post-emergence. Kaplan–Meier curves were used to estimate the adult survivor function.

The survival analysis revealed significant effects of larval diet on the adult longevity in both species (Figure 7). For GT males (Figure 7a), diet 3 yielded the longest median survival (19.5 days), significantly exceeding diet 2 (11 days, *p* = 0.0021) and diet 1 (10 days, *p* = 0.0064), with no difference between diets 1 and 2 (*p* = 0.6976). In GT females (Figure 7b), median survival increased with diet quality (diet 1, 21 days; diet 2, 30.5 days; diet 3, 37.5 days), although the differences were not statistically significant (*p* > 0.05). For AEG males (Figure 7c), diet 1 supported the longest survival (24 days), significantly outperforming diet 2 (12 days, *p* = 0.0256). Longevity on diet 3 was intermediate, with no significant differences compared with diet 1 (*p* = 0.0611) or diet 2 (*p* = 0.3816). In contrast, AEG females exhibited a different pattern: diet 2 resulted in the longest median survival (55 days), significantly exceeding diet 1 (34.5 days, *p* < 0.0001). Survival on diet 3 was intermediate, with no significant differences compared with diet 1 (*p* = 0.0537) or diet 2 (*p* = 0.2280). Overall, diet 3 consistently improved survival in GT mosquitoes, albeit with varying degrees of significance, while its effects on AEG mosquitoes were more inconsistent.

## 4. Discussion

This study demonstrated that larval diet composition shaped *Ae. albopictus* and *Ae. aegypti* fitness traits, aligning with extensive research on *Aedes* rearing [1,6,17,18]. Diet 3 (tortoise food) enhanced pupation and longevity at a lower cost (5.5 USD/kg vs. 17.8 USD/kg for diet 2), while diet 1 supported higher GT fecundity, offering options for mass rearing and colony maintenance. These observations underscore the need to balance cost, quality, and rearing objectives in SIT/IIT programs.

### 4.1. Diet Composition and Developmental Success

Diet 3’s strong performance in pupation and survival (Table 2, Figure 7) underscores the importance of macronutrient balance, proteins (≥38%), fats (≥4%), and dietary fiber (≤8%) in driving larval development. This echoes a study [17] that found that diets exceeding 35% protein significantly boosted *Ae. albopictus* pupation yields compared with lower-protein alternatives and Senevirathna et al. (2020) [19], who achieved high pupation with a cost-effective fishmeal diet. Diet 2’s consistent underperformance suggests a nutritional shortfall, possibly a deficiency in bioavailable protein despite its high content (≈50%), mirroring findings from [10], where excessive lipids impaired *Anopheles* larval survival. Although diet 1 meets IAEA standards [11], its higher cost limits scalability. The use of the aposymbiotic *Ae. albopictus* strain (GT) is an operationally justified choice for SIT-based vector control as it exhibits life history traits comparable to wild-type populations [5,13]. Critically, its *Wolbachia*-free status enables its use as a self-marking line to distinguish released males from wild populations—an important advantage for monitoring release ratios and maintaining suppression efficiency. Given its proven field performance, our evaluation of larval diets using this strain provides directly applicable insights for optimizing cost-effective rearing in SIT programs.

### 4.2. Morphological Traits as Proxies for Fitness

The pupal size and wing length, established predictors of adult fitness and competitiveness [19,20], responded markedly to diet composition (Figure 3) [6,7,19]. Diets 1 and 3 consistently outperformed diet 2, producing larger pupae and longer wings in both species, aligning with [8], who associated modest pupal size gains with enhanced *Ae. aegypti* survival. Larger female pupae under these diets may improve mechanical sex separation efficiency in SIT/IIT mass rearing [5,17,21], although our lab-scale study could not confirm this. The wing length, linked to dispersal and mating potential [9,22], suggests diets 1 and 3 could yield more field-capable males, a notion supported by a study [6] that tied wing size increases to improved flight capacity. Sex-specific patterns—males favoring diet 3 and females favoring diet 1—point to divergent nutritional needs, inviting further exploration of protein–lipid ratios [7].

### 4.3. Flight Ability and Eclosion: Functional Performance

Uniformly high eclosion rates across diets (Figure 4) indicated that baseline nutritional requirements for emergence were satisfied, consistent with [1], where even suboptimal diets sustained near-complete eclosion in *Ae. aegypti*. Male flight ability, however, revealed species-specific differences (Figure 5): diet 3 enhanced *Ae. aegypti* performance, whereas *Ae. albopictus* showed better outcomes with diet 1. These findings align with [17,18], likely reflecting species-specific metabolic requirements, and underscore the need to tailor diets to species-specific demands in SIT/IIT programs, where flight performance is pivotal for sterile male efficacy [7,23].

### 4.4. Reproductive Dynamics and Potential Life History Trade-Offs

The fecundity and longevity outcomes (Figure 6 and Figure 7) suggested diet-induced trade-offs. Diet 3 elevated both metrics in *Ae. aegypti*, while diet 1 maximized *Ae. albopictus* fecundity. Stable egg hatch rates across diets suggested viability was less diet-dependent than egg production, aligning with [14]. These patterns resonated with life history theory [24], where resource allocation may favor either fecundity or survival, although *Ae. aegypti* on diet 3 suggested nutrient density (Table 1) can sometimes support both.

### 4.5. Implication for Vector Control and Future Directions

These results offer actionable insights for *Aedes* mass rearing, with diet 3’s low cost and favorable fitness benefits outcomes suggesting significant savings over pricier commercial diets [11]. These attributes position diet 3 as a promising tool for cost-effective production, a critical factor in scaling SIT/IIT programs in resource-constrained settings [21]. Diet 1’s strength in supporting reproductive output aids colony maintenance, a priority for sustained rearing [17], while diet 2’s poor performance underscores the risks of untested formulations, as noted in a study [10]. The observed pupal sex ratios may further refine rearing strategies, informing sex separation without relying on assumed ratios [12]. However, these laboratory-derived benefits, while encouraging, require field validation to confirm their impacts on SIT/IIT outcomes, such as male competitiveness and population suppression, as demonstrated in release trials [5]. Future research should (1) evaluate these diets in large-scale facilities, (2) assess field competitiveness via mark–release–recapture studies, (3) investigate micronutrient contributions absent from Table 1, and (4) quantify trade-offs with statistical models. These efforts will bridge the lab-to-field divide, advancing sustainable control of dengue and Zika.

## 5. Conclusions

This study underscores the importance of larval diet optimization in enhancing the fitness and reproductive performance of *Ae. albopictus* and *Ae. aegypti* for mass-rearing programs [18,25]. Our analysis demonstrates that diet 3, a low-cost formulation, supports key life history traits, offering significant savings over commercial diets and potential for scaled production. Conversely, diet 1 enhances *Ae. albopictus* reproductive output critical for sustained colony maintenance. Further research should refine diet formulations and assess micronutrient roles to develop resource-efficient strategies for dengue and Zika control.

## Figures and Tables

**Table 1:** Ingredients and nutritional composition of the three larval diets.

Variable		Diet 1	Diet 2	Diet 3
Proportion		P:S:Y = 6:3:1 ^a^	B:S:Y = 6:3:1 ^b^	Tortoise food ^c^
Price		≈12.3 dollars/kg	≈17.8 dollars/kg	≈5.5 dollars/kg
Color		Light brown	Dark brown	Dark green
Texture		Powder	Powder	Particle (3 × 7 mm) ^e^
Physicochemical properties of the main component	Proteins	≈20%	≥50%	≥38%
	Fats	≈3.5%	≤8%	≥4.0%
	Dietary fiber	- ^d^	- ^d^	≤8.0%
	Ca^2+^	- ^d^	- ^d^	≤4.5%
	Total phosphorus	- ^d^	- ^d^	≥1.5%

^a^: P, pork liver powder; S, shrimp powder; Y, yeast powder. ^b^: B, bovine liver powder; S, shrimp powder; Y, yeast powder. ^c^: Tortoise food is a mature commodity made in China, which is used to feed tortoises and developed to feed mosquito larvae in this study creatively. ^d^: Unknown. ^e^: 3 × 7 mm means the size of the particle.

**Table 2:** Pupation rates (±SD) of *Ae. albopictus* and *Ae. aegypti* on days 8 and 10 post-L1 under different larval diets.

Diet	Rep	Pupation Rate on the 8th Day from L1				Pupation Rate on the 10th Day from L1			
		GT		AEG		GT		AEG	
		Male	Female	Male	Female	Male	Female	Male	Female
Diet 1	4	0.36 ± 0.04 ^a^	0.14 ± 0.02 ^a^	0.25 ± 0.08 ^b^	0.04 ± 0.03 ^b^	0.46 ± 0.05 ^a^	0.30 ± 0.04 ^a^	0.50 ± 0.07 ^a^	0.29 ± 0.03 ^a^
Diet 2	4	0.17 ± 0.01 ^b^	0.03 ± 0.01 ^b^	0.08 ± 0.02 ^c^	0.02 ± 0.02 ^b^	0.33 ± 0.03 ^b^	0.12 ± 0.01 ^b^	0.25 ± 0.02 ^b^	0.11 ± 0.04 ^b^
Diet 3	4	0.35 ± 0.08 ^a^	0.15 ± 0.02 ^a^	0.37 ± 0.03 ^a^	0.10 ± 0.03 ^a^	0.44 ± 0.06 ^a^	0.34 ± 0.05 ^a^	0.56 ± 0.02 ^a^	0.33 ± 0.03 ^a^

Within each column, values followed by different lowercase were statistically different using ANOVA analysis and the Tukey post hoc test (*p* < 0.05). Data are presented as mean ± SD.

**Table 3:** Male pupal recovery rates (±SD) of *Ae. albopictus* and *Ae. aegypti* on days 8 and 10 post-L1 under different larval diets.

Strain	Diet	Rep	On the 8th Day		On the 10th Day	
			Total Pupa Collected (n)	Recovery of Male Pupae	Total Pupa Collected (n)	Recovery of Male Pupae
GT	Diet 1	4	35.75 ± 4.19 ^a^	0.72 ± 0.04 ^b^	45.50 ± 4.93 ^a^	0.60 ± 0.05 ^a^
	Diet 2	4	17.00 ± 1.41 ^b^	0.86 ± 0.03 ^a^	33.25 ± 2.99 ^b^	0.74 ± 0.04 ^b^
	Diet 3	4	35.25 ± 8.14 ^a^	0.70 ± 0.08 ^b^	43.75 ± 5.50 ^a^	0.57 ± 0.06 ^a^
AEG	Diet 1	4	24.75 ± 8.38 ^a^	0.89 ± 0.05 ^a^	50.25 ± 6.65 ^a^	0.63 ± 0.06 ^a^
	Diet 2	4	7.50 ± 2.38 ^a^	0.84 ± 0.13 ^a^	24.75 ± 1.89 ^a^	0.70 ± 0.06 ^a^
	Diet 3	4	37.00 ± 3.27 ^a^	0.78 ± 0.06 ^a^	56.25 ± 1.71 ^a^	0.63 ± 0.03 ^a^

Within each column, values followed by different lowercase letters were statistically different using ANOVA analysis and the Tukey post hoc test (*p* < 0.05). Data are presented as mean ± SD.

## Data Availability

The original contributions presented in this study are included in the article. Further inquiries can be directed to the corresponding authors.
